# Conserving Eyesight

**Published:** 1917-06

**Authors:** D. E. Compere

**Affiliations:** Dallas, Texas


### CONSERVING EYESIGHT

BY D. E. COMPERE, M. D., DALLAS, TEXAS.

We are living in an age of conservation. We are ever striving to conserve time, strength, energy, and even life itself.

The varied forms of conservation deserve much commendation, but why not conserve one of our most valuable possessions—eyesight? Why are there more people wearing glasses today than in any age in the history of the world? Simply because we are deviating farther from nature than ever before, taxing the eyes more and more, and as the result we are having to pay the penalty of weak, strained eyes, blurred vision, headaches, reflexes and glasses. A glass to the eye may be classed as a brace to a sprained ankle, to relieve the strain. Our forefathers never worked over small, close figures, under strong artificial illumination for hours, days and years, as we do. They knew nothing of the constant strain of the moving picture show, nor the bright glare, sunshine and sand from an automobile, as well as many, many strenuous tasks we impose on our eyes every day, and think nothing of it. As civilization advances, the strain on the eye increases. One marked example of this fact is attested by the movies. Yet a picture is one of the most instructive methods of education we have today, as *it speaks all languages.* Therefore, as the demands on the eye will never diminish, we must recognize the necessity of overcoming the strain which produces the defects.

The eye is a dark chamber with a series of convex refracting surfaces and certain intraocular media or watery substances. The sense of sight consists of three visual perceptions or subsenses, viz.: Light, sense, color sense and form sense. The field of vision is divided, first, into central or direct, which is to fix the eye on one object and see clearly and distinctly. Second, peripheral, or indirect vision, is to see around one point and see objects not looked at, which are less distinct or a duller sensation.

The evil effects of the constant strain and excessive demands upon the muscles of accommodation, to exclude this indirect vision, to the extent of preventing it from interfering with the direct vision, are manifested by spasm or cramp, which produces blurred or indistinct vision. After continuing this over-taxing, say, for distinct objects, the eye uses up part of its accommodative energy, which actually leaves less at disposal for near objects. In extreme cases of high degree accommodative spasm, serious errors might arise if this cramp is overlooked, and often a person cannot maintain a sustained view of an object at any distance without suffering pain in and around the eyes.

Science, no matter how scientific, benefits humanity little, unless its findings can be crystallized into practical application. Therefore, by harnessing up this indirect vision when not needed, and relieving the eyes of a useless expenditure of accommodative power, we will render them more capable of producing even extremely strenuous work with less ill results.

I have a devise which is a mechanical concentrator, and will relieve the muscular spasm in the act of accommodation. It consists of a cup-shaped mounting or goggles that fit close around the eyes so as to exclude all light, with an adjustable diaphragm before each eye.

Numerous letters of inquiry and indorsement have been received from all parts of the United States. Also one from Manila, Philippine Islands. Another from Bridgetown, Barbados, British West Indies.

